# Direct comparison of the tofu-like precipitate formation by adding different coagulants: magnesium chloride and glucono-δ-lactone

**DOI:** 10.1016/j.heliyon.2021.e07239

**Published:** 2021-06-08

**Authors:** Yasuhiro Arii, Yoshinori Sano, Kaho Nishizawa

**Affiliations:** a Department of Innovative Food Sciences, School of Food Sciences and Nutrition, Mukogawa Women's University, Nishinomiya, Hyogo, 663-8558, Japan; b Research Institute for Nutrition Sciences, Mukogawa Women's University, Nishinomiya, Hyogo, 663-8558, Japan

**Keywords:** Glucono-δ-lactone, Magnesium chloride, Soymilk, Tofu, Urea soluble protein

## Abstract

Tofu is produced by adding a coagulant such as MgCl_2_ and glucono-δ-lactone (GDL) in soymilk. However, the molecular mechanism of tofu formation by adding these coagulants has been compared between the results obtained under different conditions. In this study, the formation of a tofu-like precipitate (TLP) by adding GDL was directly compared with that formed by adding MgCl_2_ under the same conditions except for the coagulants. The effects of both the coagulants were almost the same on the changes in the precipitate weight, supernatant protein concentration, and urea-soluble protein concentration, indicating that a common contributing factor induces TLP formation. However, the effects of the coagulants on pH were largely different, suggesting that pH reduction is not an absolute requirement in TLP formation induced by adding MgCl_2_. Moreover, the findings of this study revealed that the decrease in the surface charge of soymilk proteins is a common initiation factor for TLP formation, whereas the intermolecular hydrophobic interaction is an important factor for the formation of urea-insoluble precipitates. Overall, these findings will be useful in discovering new coagulants to enhance the quality characteristics of tofu.

## 1 Introduction

Tofu is a soybean product that has gained global popularity for its rich nutritional value. It is produced by coagulating soymilk with different coagulants, including magnesium chloride (MgCl_2_), calcium sulfate (CaSO_4_), and glucono-δ-lactone (GDL). MgCl_2_ and CaSO_4_ are the inorganic salts widely used as coagulants to produce tofu with a long history. In addition to Ca^2+^ and Mg^2+^, several other multivalent metal ions have also been used to produce a tofu-like precipitate (TLP) (Arii and Takenaka, 2014). In a previous study, we have shown that metal ions act as the initiators of protein association in tofu formation (Arii and Takenaka, 2014); however, their role as linkers to interact with the carboxyl groups of soymilk proteins is plausible (Tamura, 1959; Appurao and Narasinga Rao, 1975; Sakakibara and Noguchi, 1977; Torikata et al., 1987; Kao et al., 2003; Arii and Takenaka, 2014; Zhao et al., 2016). Furthermore, in our previous study, the formation of silken and regular tofu with a smooth precipitate and a rough precipitate was demonstrated to be dependent on the low and high concentration of coagulants, respectively. It has also been shown that the smooth and rough precipitates are mainly composed of urea-soluble precipitates (USPs) and urea-insoluble precipitates (UIPs), respectively (Arii and Takenaka, 2013). USPs and UIPs have different solubilities in 2 M urea, surface charges, and water contents, and the transition point of the USP to the UIP formation has been shown to be a useful indicator of the TLP formation to elucidate the molecular mechanism relevant to tofu formation. The evidence represents that TLP is largely separated into USP and UIP by adding MgCl_2_ in different concentrations.

In addition to metal ions, GDL is also used as a coagulant for the industrial processing of tofu, though with a comparatively shorter history than metal ions (Nakayama et al., 1965; Hsia et al., 2016; Zhao et al., 2018; Jun et al., 2019). GDL, an acidic coagulant, is spontaneously hydrolyzed in water to form gluconic acid (Sawyer and Bagger, 1959); therefore, reducing the pH (Chen et al., 2016). Consequently, the pH reduction leads to TLP formation (Arii and Nishizawa, 2020) by decreasing the electrostatic repulsion between soymilk proteins during the tofu formation (Cavallieri & da Cunha, 2008). However, tofu produced by adding GDL is harder than those produced by adding CaSO_4_ (Guo and Ono, 2005). The yield was also higher with GDL addition than that with CaSO_4_ addition (Shen1991).

To date, most of the reported studies on tofu formation, including the direct comparative studies that compared the results obtained under different conditions (Guo and Ono, 2005; Shen et al., 1991), have focused on the textural and yield properties of tofu, as these properties are important for consumer preference and marketability of tofu. In addition, it has been reported that the tofu processing conditions influenced the storage proteins responsible for the textural properties of tofu (Syah et al., 2015). Therefore, we speculated that direct comparison of tofu formation under the same processing conditions could provide insights into the molecular mechanism of tofu formation and the effect of coagulants. Nevertheless, very few direct comparative studies have investigated the molecular mechanism of tofu formation and the effects of coagulants. For instance, a previous report has described that the addition of calcium ions induces pH reduction to form a network between proteins (Ono et al., 1993), wherein another report has described that GDL also induces pH reduction to form a network between proteins (Cavallieri & da Cunha, 2008). Linking these studies, it can be inferred that the reduction in pH is an important factor for TLP formation; however, it warrants validation through in-depth analyses.

Therefore, in the present study, we directly compared the molecular mechanisms underlying TLP formation using MgCl_2_ and GDL as coagulants. In addition, the effects of these coagulants on USP and UIP formation were also investigated. The direct comparison revealing the key factors regulating the TLP formation could be useful for discovering new coagulants.

## 2 Materials and methods

### 2.1 Materials

Soymilk (50 g protein/L) was purchased from Sujahta Meiraku (Nagoya, Aichi, Japan). The protein assay dye reagent was purchased from Bio-Rad Laboratories (Hercules, CA, USA). GDL was generously provided by Ako Kasei Co. Ltd. (Ako, Hyogo, Japan). Other reagents were purchased from FUJIFILM Wako Pure Chemical Corporation (Osaka, Japan).

### 2.2 Preparation of precipitates and supernatants from soymilk

Precipitates and supernatants were prepared from soymilk according to the methods previously described by previous studies (Arii and Takenaka, 2013; Yang and James, 2013; Arii and Nishizawa, 2020), with minor modifications. Soymilk was incubated at 85 °C for 10 min MgCl_2_ and GDL were dissolved in distilled water and used as coagulant solutions at various concentrations (0–200 mM). A 0.11 volume of each coagulant solution was added to the incubated soymilk, mixed well, and reincubated at 85 °C for 60 min (Hsia et al., 2016). The mixture was then incubated on ice for 15 min and precipitated by centrifugation at 4,100 × *g* for 10 min at 4 °C to separate the supernatant. The precipitate was used to determine the precipitation efficiency and urea solubility of proteins in the precipitates, and the supernatant was used to determine the protein concentration. Moreover, the wet precipitate weight and supernatant protein concentrations were investigated for the coagulant concentrations.

### 2.3 Determination of the precipitation efficiency

The weight of the wet precipitate was measured according to the method described by Arii and Takenaka (2013). The precipitation efficiency was expressed as the weight of the wet precipitate relative to that of the initial soymilk. Data are presented as the mean ± standard deviation (SD) of three independent experiments.

### 2.4 Extraction of urea-soluble proteins (USPs)

USPs were extracted from the precipitates following the method described in our previous studies (Arii and Takenaka, 2013; Arii and Nishizawa, 2018). Briefly, the precipitate was suspended in 2 M urea in a volume 10 fold that of the initial soymilk, incubated at 50 °C for 60 min, and agitated every 15 min with a vortex mixer. After incubation, the suspension was separated into a supernatant and precipitated by centrifugation at 4,100 × *g* for 10 min at 4 °C. The concentration of USPs in the supernatant was determined as described below.

### 2.5 Determination of the supernatant protein concentration

Proteins in the supernatants were quantified by the Bradford method using a protein assay dye reagent concentrate (Bio-Rad Laboratories). The residual protein concentration ratio was expressed as the protein concentration in the supernatant after coagulant addition to that of soymilk diluted 10:1 in distilled water. The USP concentration ratio was expressed as the concentration of USPs to the protein concentration of soymilk. Data are presented as the mean ± SD of three independent experiments.

### 2.6 Determination of midpoint values

The midpoint concentration and pH values were determined from the plots for the supernatant protein concentration using a fitting analysis with sigmoidal function using KaleidaGraph 4.5 software (Synergy Software, PA, USA).

## 3 Results

### 3.1 TLP formation by adding MgCl_2_ and GDL

**Figure 1:**
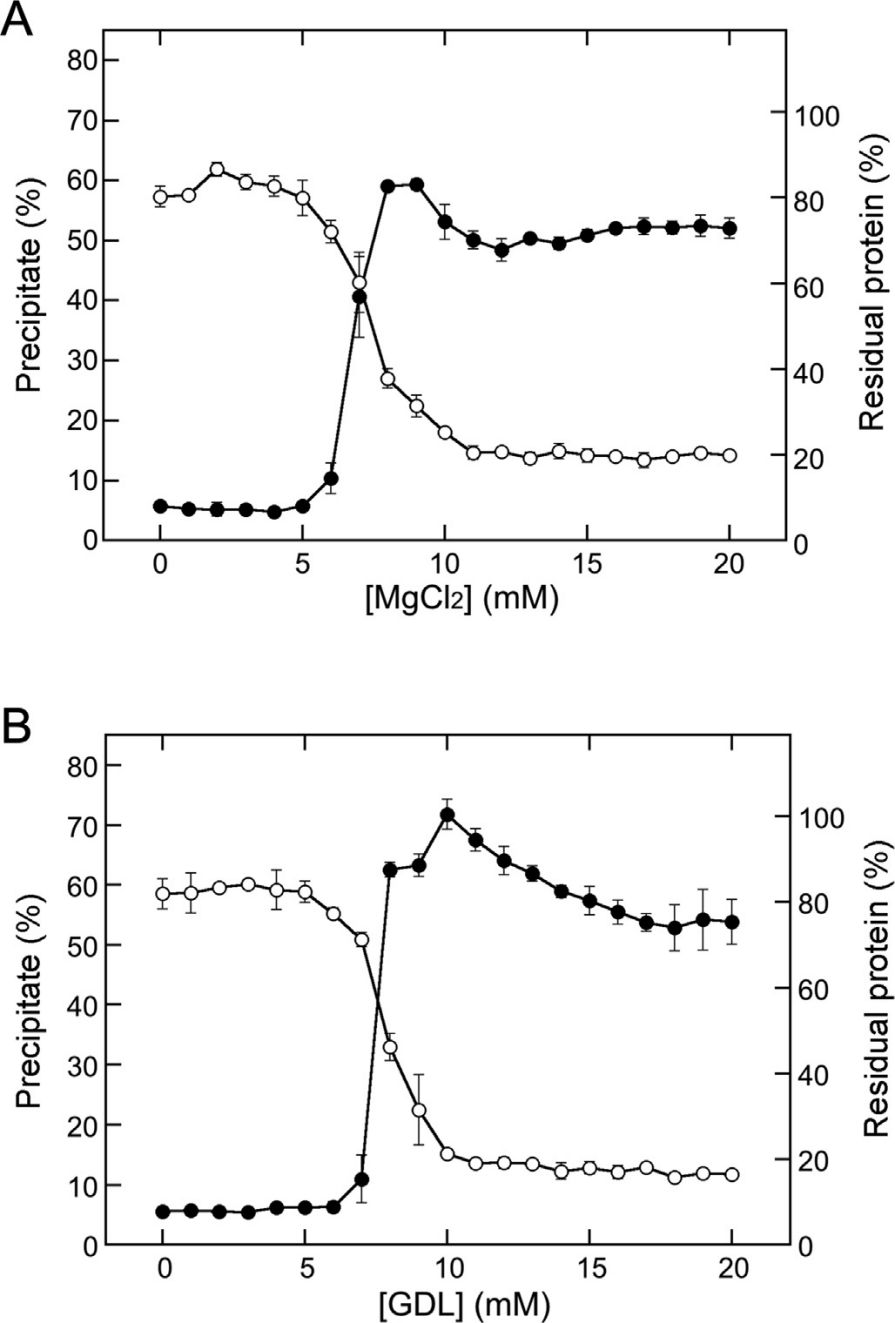
Changes in precipitate formation by adding different coagulants. MgCl_2_ (A) and GDL (B) were added to soymilk at the indicated concentrations. Open and closed circles represent residual protein concentration and precipitation efficiency, respectively. The precipitation efficiency is expressed as the percentage of the wet weight to the initial weight of soymilk. The data are shown as the mean ± standard deviation (SD) of three independent experiments.

For the comparison of TLP formation, MgCl_2_ and GDL were added to soymilk at concentrations ranging from 0 mM to 20 mM (Figure 1). The findings showed that with MgCl_2_ addition, the precipitation efficiency sharply increased (from 10 to 60%) at the concentration range of 6–8 mM MgCl_2,_ followed by a short plateau (60%) at the concentration of 9 mM, decreased (to 50%) at a concentration of 11 mM, and then remained at almost the same level (Figure 1A). The observed changes in precipitation efficiency could be attributed to the water content of the TLPs (Arii and Takenaka, 2013). In contrast, the supernatant protein concentrations significantly decreased with increasing MgCl_2_ concentration and almost precipitated at concentrations greater than 11 mM (Figure 1A). The midpoint concentration for the reduction of supernatant proteins was 7.4 mM (*R*^2^ = 0.997), as observed from the supernatant protein concentration plot. These behaviors are consistent with those reported in our previous reports (Arii & Takenaka, 2013, 2014; Arii and Nishizawa, 2018). With GDL addition (Figure 1B), the precipitation efficiency sharply increased in a concentration of 7–8 mM from 10 to 60%, followed by a further increase to reach a peak of approximately 70% at a concentration of 10 mM, then, the weight gradually decreased to approximately 50% in the concentration range of 10–17 mM, and then remained at almost the same level. The decrease might also have arisen from the different water contents. Furthermore, the findings show that the supernatant proteins were almost precipitated at concentrations greater than 10 mM (Figure 1B), and the midpoint concentration value for the reduction of supernatant proteins was 7.9 mM (*R*^2^ = 0.999). These results showed that GDL addition achieved a higher peak than that obtained by MgCl_2_ addition and revealed a gradually decreasing trend after the peak in weight, a characteristic feature of GDL addition. Interestingly, the midpoint concentrations in both cases were almost the same; however, this consensus may be incidental. Nevertheless, these changes can be stoichiometrically compared in the same coagulant concentration range.

### 3.2 Changes in the USP concentration with MgCl_2_ addition and GDL addition

**Figure 2:**
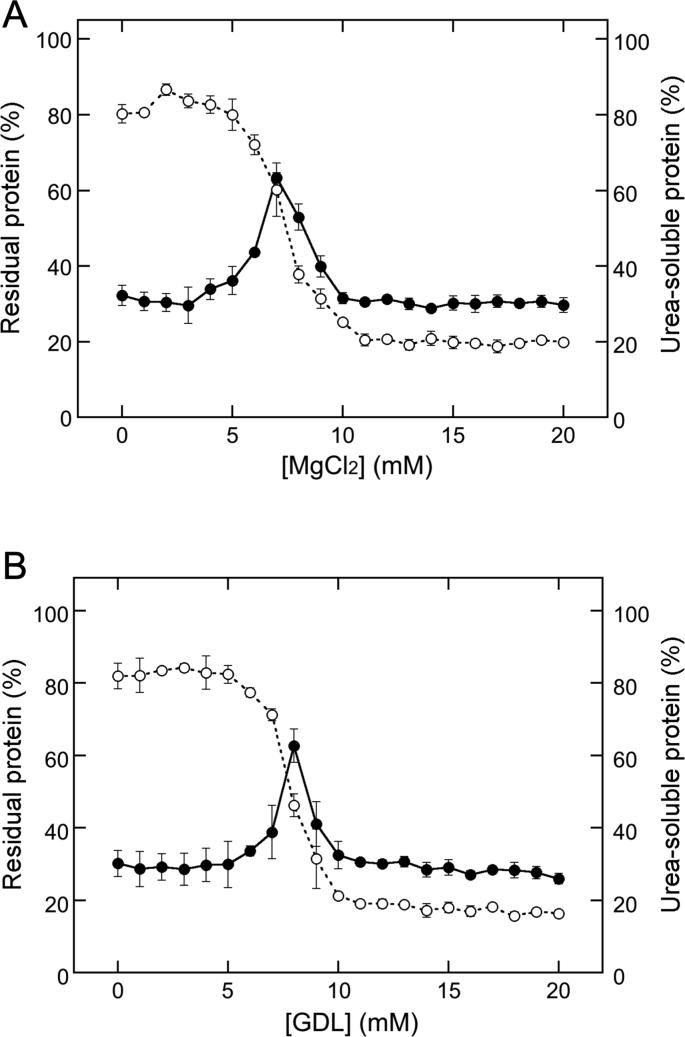
Changes in urea-soluble protein (USP) concentrations in the presence of MgCl_2_ and GDL. MgCl_2_ (A) and GDL (B) were added to soymilk at the indicated concentrations. Open circles and dotted lines represent the residual protein concentration shown in Figure 1. Closed circles and solid lines represent USP concentrations. The data are shown as the mean ± SD of three independent experiments.

Previous studies have shown that USP and UIP vary with the concentrations of MgCl_2_ in TLP formation (Arii and Takenaka, 2013; Arii and Nishizawa, 2018). As described in a previous study, the decrease in USP represents the formation of UIP, and the peak shows the transition point from the USP formation to UIP formation (Arii and Nishizawa, 2018). At a concentration of 7 mM, the transition from USP formation to UIP formation was distinct (Arii and Nishizawa, 2018). To compare the transitions in different coagulant additions, TLPs were resuspended in 2 M urea and then separated into precipitate and supernatant, then the supernatant protein concentrations were determined (Figure 2). USP increased with increasing MgCl_2_ concentrations up to 7 mM and then decreased (Figure 2A). The USP concentration decreased from approximately 60% (at 7 mM MgCl_2_) to 30% (at 10 mM MgCl_2_) and then remained at almost the same level, indicating 7 mM MgCl_2_ as the transition point (Figure 2A). In addition, USP concentration increased with increasing GDL concentration in the range of 0–8 mM, decreased to approximately 30% at a concentration of 10 mM, and then remained at almost the same level. Based on the findings of our previous study (Arii and Nishizawa, 2018), this behavior indicates that the transition from USP formation to UIP formation is also induced by increasing GDL concentration, and the transition point was 8 mM, which was slightly higher than that in MgCl_2_ addition. A similar trend of USP and UIP formation with MgCl_2_ and GDL implies that the mechanism of TLP formation could be the same with different coagulants.

### 3.3 Changes in the appearance of TLP with differing USPs

**Figure 3:**
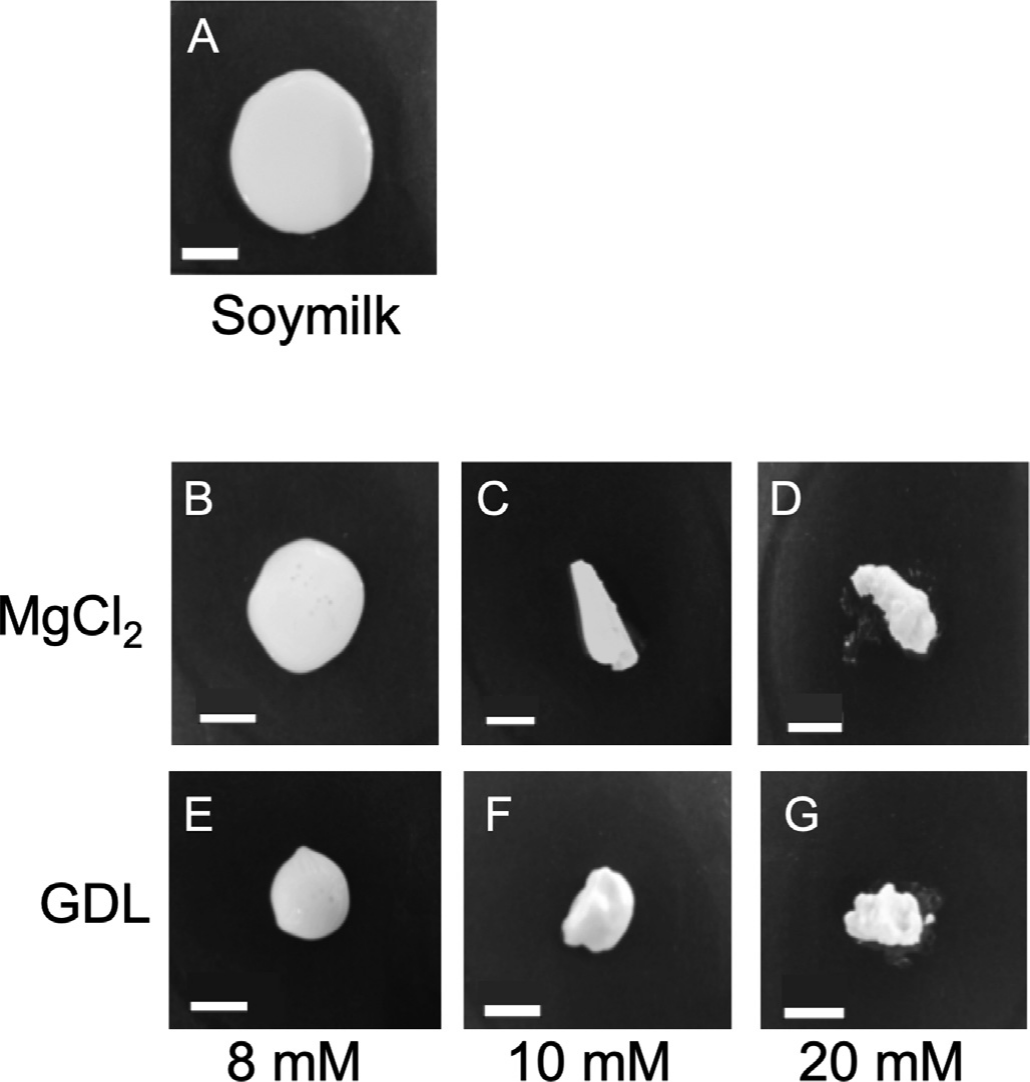
Appearances of the precipitate by adding coagulant at final concentrations. (A) The aspect of soymilk is represented in an initial volume (0.9 mL). MgCl_2_ (B–D) and GDL (E–G) were added to soymilk at the final concentration of 8 mM (B and E), 10 mM (C and F), or 20 mM (D and G). Bars represent 1 cm.

A previous study reported that the appearance of TLP changes with different MgCl_2_ concentrations —TLP has a smooth surface at lower MgCl_2_ concentrations and a rough surface at higher MgCl_2_ concentrations (Arii and Takenaka, 2013). Here, we compared the visible appearance of TLP formed with the addition of GDL or MgCl_2_ as coagulants (Figure 3). The physicochemical characteristics of TLP were estimated using different concentrations of the coagulants (8, 10, and 20 mM). At a concentration of 8 mM, both TLPs contained high amounts of USP (Figure 2) and precipitated with a smooth surface (Figure 3B and 3E), whereas at a concentration of 10 mM, both TLPs contained high UIP (Figure 2). Although the UIP formation was of the same degree, the weight of the resultant TLP differed (Figure 1). At a concentration of 10 mM, the weight was decreased to almost 50% with MgCl_2_ addition while reached the peak with GDL addition. At this concentration, the precipitates had a rough surface with MgCl_2_ addition (Figure 3C) and a smooth surface with GDL addition (Figure 3F). These results indicated that the UIP might not be the key factor regulating the texture of TLP. Moreover, at a concentration of 20 mM, both TLPs precipitated with a rough surface (Figure 3D and 3G), and at this concentration, both TLPs had the same weight and were rich in UIP (Figures 1 and 2). The results indicate that TLP has a smooth surface at a lower coagulant concentration and a rough surface at a higher coagulant concentration in both coagulant additions.

### 3.4 Association between pH change and TLP formation

**Figure 4:**
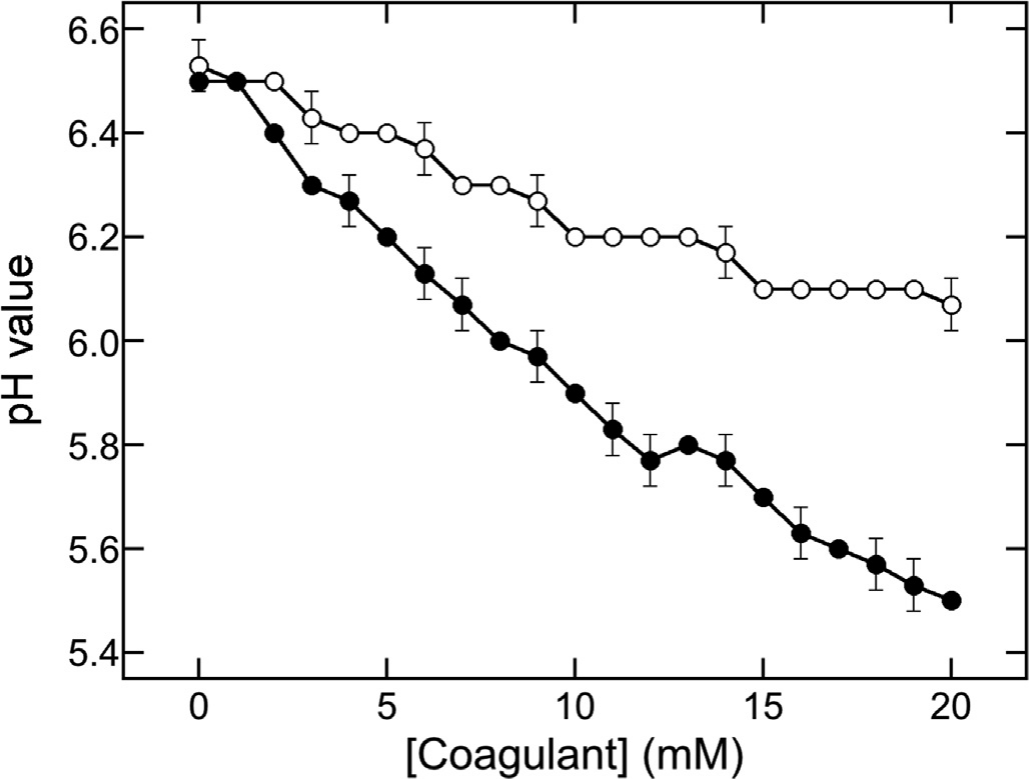
Changes in pH values in the presence of MgCl_2_ and GDL. MgCl_2_ (open circles) and GDL (closed circles) were added to soymilk at the indicated concentrations. The data are shown as mean ± SD of three independent experiments.

Figures 1 and 2 show that the coagulant concentrations ranging from 0 to 20 mM affected TLP formations. Therefore, we tested the effects of MgCl_2_ and GDL additions on the pH of the supernatants at these concentrations (Figure 4). The pH values decreased with an increase in the concentration of MgCl_2_ and GDL, whereas the rate of decrease by GDL addition was higher than that by MgCl_2_ addition.

**Figure 5:**
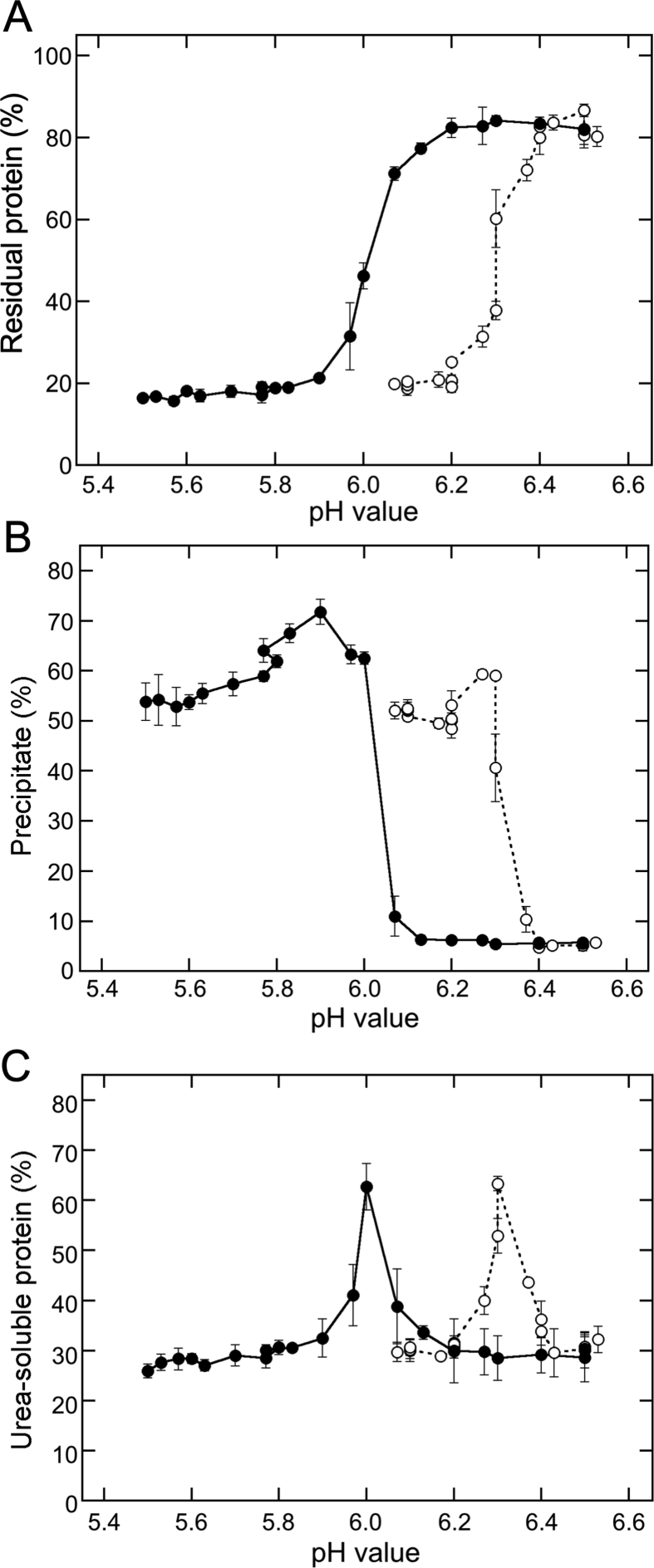
Comparison of the precipitate formation between MgCl_2_ and GDL addition in pH value. Precipitation efficiency (A), residual protein concentrations (B) and USP concentrations (C) were plotted against the pH value in the presence of MgCl_2_ (open circles) or GDL (closed circles). The data obtained for the precipitation efficiency and residual protein concentrations (Figure 1), the USP concentrations (Figure 2), and the pH value (Figure 4) were used to construct the plot.

Next, we investigated the association between the changes in pH and TLP formation by plotting the data shown in Figures 1 and 2 against the pH values shown in Figure 4; the resultant plot is shown in Figure 5. The wet precipitate weight was increased at a pH of less than 6.4, with MgCl_2_ addition and a pH value of less than 6.1, with GDL addition (Figure 5A). However, a slight decrease was observed at a pH value of 6.2, with MgCl_2_ addition and at a pH value of 5.8, with GDL addition. Though the observed changes in weight were similar, the pH values inducing the changes were largely different. These results suggested that the pH value might not be related to the formation of TLP by adding MgCl_2_.

Furthermore, the changes in pH revealed a sigmoidal pattern of association with the concentrations of the supernatant proteins in response to both the coagulants (Figure 5B). After a continuous decline, the protein concentration reached the same level at a pH value of less than 6.2, with MgCl_2_ addition, and at a pH value of less than 5.8, with GDL addition. The midpoint pH value was determined to be 6.3 for MgCl_2_ addition (*R*^2^ = 0.979) and 6.0 for GDL addition (*R*^2^ = 0.999) from the plots. Though the changes in the protein concentration and the wet precipitate weight with both coagulants were similar, the pH values differed markedly.

In the USP formation (Figure 5C), the USP concentration reached the peak at a pH value of 6.3, with MgCl_2_ addition and at a pH value of 6.0, with GDL addition, indicating that the transition from USP formation to UIP formation is not related to the pH value. From these results, it was clear that the pH reduction is not related to TLP formation while using MgCl_2_ as the coagulant; in other words, pH reduction is not an absolute requirement for TLP formation.

## 4 Discussion

**Figure 6:**
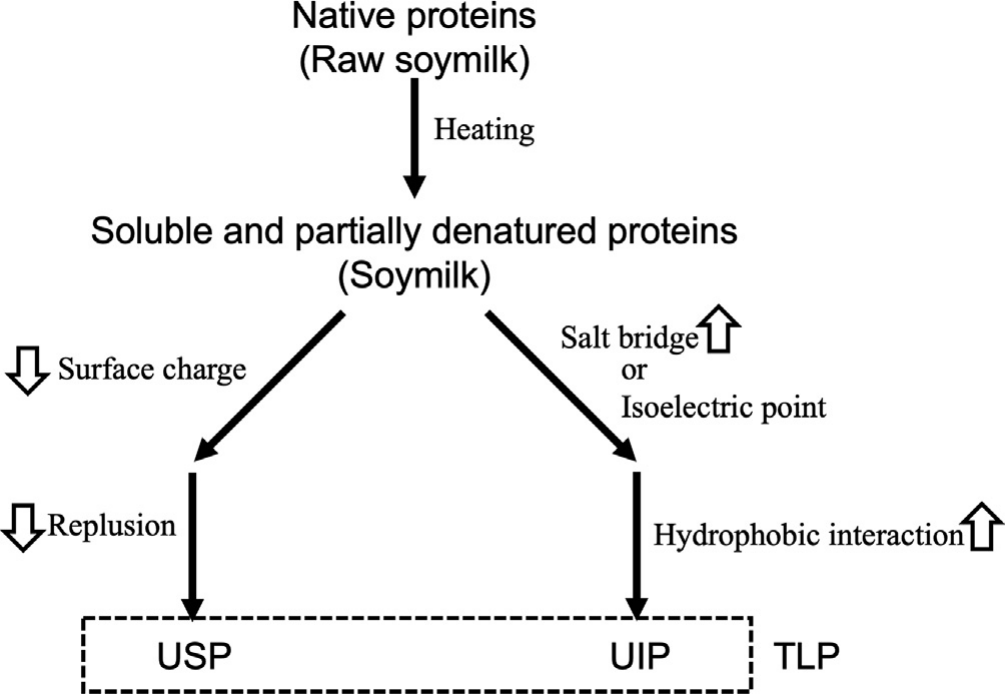
Schematic presentation of the USP and UIP formation during TLP formation.

This study demonstrated that the transition from USP formation to UIP formation depended on the concentration of coagulants (Figure 2). The effects of GDL and MgCl_2_ addition were similar on the precipitate weight, supernatant protein concentration, and USP concentration changes (Figures 1 and 2). These similarities show the existence of a common mechanism for the formation of TLP in the addition of MgCl_2_ and GDL. Furthermore, the findings also demonstrate that TLP is largely separated into USP and UIP by adding coagulants in different concentrations. Based on these results, we proposed a scheme for the TLP formation (Figure 6).

The present study demonstrated similar behaviors of the TLP formation by MgCl_2_ addition as those reported in our previous study (Arii and Nishizawa, 2018), suggesting that for the USP formation, it is an important factor to decrease the repulsion following a decrease in the surface charge on soymilk proteins by MgCl_2_ addition and GDL addition (Figure 5), whereas for UIP formation, the increase in hydrophobic interactions between soymilk proteins is an important factor (Arii and Takenaka, 2013; Arii and Nishizawa, 2018). The increase in hydrophobic interactions is induced by the proximity of soymilk proteins. The proximity is generated from the formation of salt bridges by MgCl_2_ addition (Arii and Takenaka, 2013) or the achievement of the isoelectric point by GDL addition (Figure 5). Salt bridges are formed through metal ions at the carboxy groups of proteins (Tamura, 1959; Appurao and Narasinga Rao, 1975; Sakakibara and Noguchi, 1977; Torikata et al., 1987; Arii and Takenaka, 2014), which decreases the surface charge of proteins. These studies indicate that the added coagulant category is inconsequential for the mechanism of TLP formation, and the reduction of the surface charge is a common inducing factor for TLP formation, representing a helpful tool for the discovery of new coagulants in tofu processing.

Previous studies have described that TLP formation is induced by achieving the isoelectronic point of soymilk proteins in GDL addition (Cavallieri & da Cunha, 2008; Ringgenberg et al., 2013), CaSO_4_ addition (Ono et al., 1993) and other organic acid coagulants (Cao et al., 2017; Sitanggang et al., 2020). In the present study, direct comparisons between MgCl_2_ and GDL addition indicated that the TLP formation with GDL addition is induced by the achievement of the isoelectronic point, which has been reported to be 5.8 (Ringgenberg et al., 2013); however, the TLP formation is not related to the isoelectronic point when MgCl_2_ is used as the coagulant (Figure 5). Moreover, it also demonstrated that the pH reduction was not directly related to the transition from USP formation to UIP formation when MgCl_2_ was added as a coagulant (Figure 5C). Taken together, these results imply that reducing the surface charge of proteins is an important factor for TLP formation, regardless of the procedure used to decrease the surface charge.

The TLP formation with GDL addition indicates that an increase in hydrogen ions generates both USP and UIP (Figures 4 and 5). Hydrogen ions are equally monovalent cations, such as sodium ions. However, sodium ions have no potential for UIP formation even in a similar concentration range (Arii and Nishizawa, 2018). Sodium ions have a larger effective ion radius than hydrogen ions (Shannon, 1976), suggesting that the different UIP formation potentials of sodium and hydrogen ions could be attributed to their ion radii. Moreover, starch hydrolysate compounds retard protein gelation in tofu processing and work as a steric hindrance interrupting the formation of protein-protein interactions (Jiang et al., 2020). In the TLP formation, the physical obstacles may also constitute a limiting factor of the proximity between the hydrophobic regions of proteins. Overall, these results indicate that the increased intermolecular hydrophobic interaction could also be an important factor for UIP formation. However, with MgCl_2_ addition, the pH of the soymilk supernatant was reduced because of the increased protein precipitation (Figures 4 and 5), indicating that under the condition of a constant pH, adding divalent cations might form new tofu-like foods with textures and tastes different from tofu. Therefore, in future studies, the effect of different divalent cations under the condition of a constant pH should be investigated to explore potential coagulants without the adverse effects on the texture and quality of tofu.

In conclusion, the proximity between proteins is an important factor in TLP formation, and the procedure for proximity is sequential. The decrease in the surface charge of proteins is a common initiation factor for TLP formation. In addition, the increase in intermolecular hydrophobic interactions is also an important factor in the formation of UIP. Overall, the common initiation factor identified here could be a helpful tool for discovering new coagulants in tofu processing; therefore, it would benefit the tofu industry to produce tofu with improved quality and enhanced consumer acceptability.

## Declarations

### Author contribution statement

Yasuhiro Arii: Conceived and designed the experiments; Performed the experiments; Analyzed and interpreted the data; Contributed reagents, materials, analysis tools or data; Wrote the paper.

Yoshinori Sano: Analyzed and interpreted the data; Wrote the paper.

Kaho Nishizawa: Performed the experiments; Contributed reagents, materials, analysis tools or data.

### Funding statement

This work was supported by JSBBA Academic Industrial R&D Support for Small-to-medium sized Enterprises.

### Data availability statement

Data will be made available on request.

### Declaration of interests statement

The authors declare no conflict of interest.

### Additional information

No additional information is available for this paper.
